# Impact of ambient sound on risk perception in humans: neuroeconomic investigations

**DOI:** 10.1038/s41598-021-84359-7

**Published:** 2021-03-08

**Authors:** Elise Payzan-LeNestour, Lionnel Pradier, James Doran, Gideon Nave, Bernard Balleine

**Affiliations:** 1 University of New South Wales Business School, Sydney, Australia; 2 The Wharton School of the University of Pennsylvania, Philadelphia, PA USA; 3 School of Psychology, University of New South Wales, Kensington, Australia

**Keywords:** Psychology, Human behaviour

## Abstract

Research in the field of multisensory perception shows that what we hear can influence what we see in a wide range of perceptual tasks. It is however unknown whether this extends to the visual perception of risk, despite the importance of the question in many applied domains where properly assessing risk is crucial, starting with financial trading. To fill this knowledge gap, we ran interviews with professional traders and conducted three laboratory studies using judgments of financial asset risk as a testbed. We provide evidence that the presence of ambient sound impacts risk perception, possibly due to the combination of facilitatory and synesthetic effects of general relevance to the perception of risk in many species as well as humans. We discuss the implications of our findings for various applied domains (e.g., financial, medical, and military decision-making), and raise new questions for future research.

## Introduction

The psychophysics literature on multisensory perception provides ample evidence that sound alters the perception of visual events in diverse contexts, even in spatial tasks where vision is expected to dominate the other senses, and in a variety of ways, including their temporal resolution^1^, intensity^2^, and motion^3^. In terms of underlying mechanisms, there is ample evidence that auditory signals are effective at “startling” decision-makers^4–6^, and that the presence of noise elicits heightened arousal in animals^7^, which in turn has been shown to facilitate information processing and behavioural responses through stimulation of the sympathetic nervous system^8^. Alongside such general facilitatory effects, evidence has emerged for “synesthetic congruency effects” whereby the introduction of a congruent auditory stimulus disambiguates the perception of an ambiguous visual stimulus^9,10^.

Therefore, there clearly is a sense in which “*what we see is what we hear*”^11^. In this paper, we bring together these insights and the neuroeconomics literature to ask if this extends to the visual perception of risk generally, and of financial risk (“volatility”) in particular. There is an urgent need to answer this question, given the importance of properly tracking risk in varying applied domains of decision-making under uncertainty, starting with financial trading. The way professional traders reportedly ‘*seek the buzz*’ to better “feel risk”^12^ suggests they guess the answer to the question is positive. Nevertheless, it is a question that has yet to be addressed scientifically.

To fill this knowledge gap, we propose and test the “Bimodality of Risk Perception Hypothesis” (henceforth, BRPH) according to which the presence of ambient sound sharpens the visual perception of extreme risk owing to the combination of the aforementioned facilitatory and synesthetic congruency mechanisms. While arousal-related facilitation is generic, as stressed above, the synesthetic congruency aspect would come from the fact that in many environments, visual and auditory components greatly vary over time and tend to covary: high (resp. low) visual volatility often comes with a high (resp. low) volume auditory background. Real-world trading floors are a case in point: Turbulent times feature both high asset price volatility, displayed in real time on trader terminal screens, and high ambient noise, whereas low volatility is usually associated with low trading activity and quiet on the floor^13,14^. Given such systematic covariation, what makes agents regard their environment as more or less risky may not be the degree of visual instability alone but rather the combination of visual instability and auditory volume.

Both the facilitatory and synesthetic congruency effects postulated by BRPH are highly plausible in light of the foregoing literature, yet hitherto untested for the visual perception of risk, despite the importance of the topic for our understanding of decision-making under uncertainty. The present study sets out to do this.

**Figure 1:**
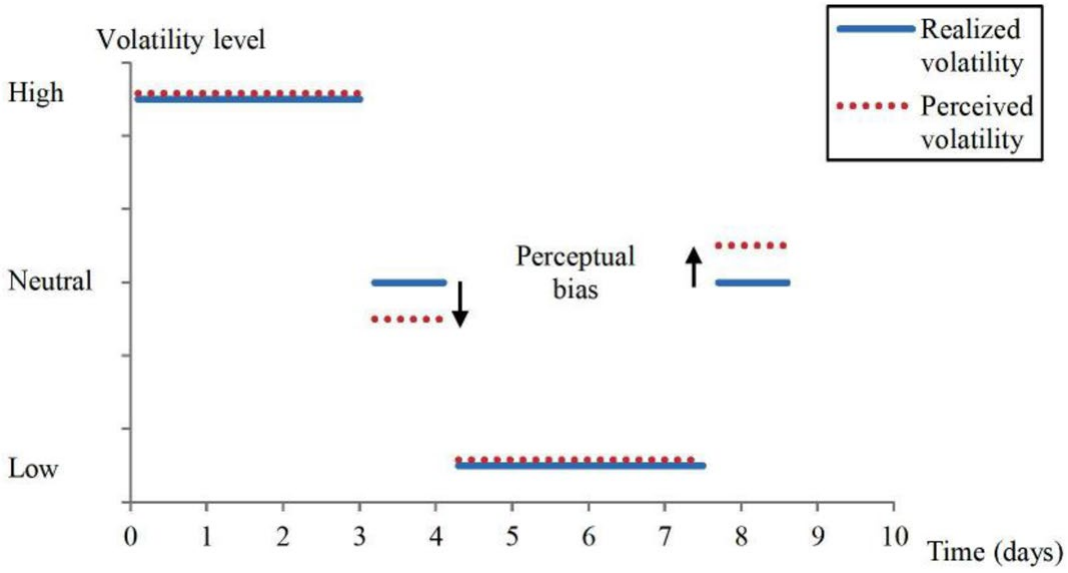
Risk after-effect in the aftermath of a volatility regime in a given financial market. The diagram is a stylized representation of a high volatility regime (top left blue horizontal line), low volatility regime (bottom blue horizontal line), and corresponding transition phase (blue horizontal line following each regime). Recent studies show that perceived volatility (dashed lines) is biased downward (resp. upward) in the aftermath of a high (resp. low) volatility regime^15,16^.

Testing BRPH appears difficult at first inasmuch as the first strategy that comes to mind to test it, namely comparing judgments of extreme visual volatility with vs. without the presence of sound, is problematic (see “Methods” for details). However, focusing on the case of financial trading allows precisely for such a test because financial markets feature prolonged phases of extreme—very high or very low—volatility (the so-called “volatility regimes”), followed by periods of intermediate, or medium, volatility (“the transition phase”). After prolonged exposure to high volatility, the agent is adapted to high volatility and hence perceives medium volatility as lower than actual (and vice versa), in what has been called “the risk after-effect” (Fig. 1). The more extreme the volatility levels during the regimes, the stronger the subsequent after-effect during the transition phase. BRPH predicts that by enhancing the perception of extreme volatility, the pairing of high (/low) volatility with high (/low) ambient noise prevailing in real-world trading floors is equivalent to making the volatility levels even more extreme during the regimes, compared to the hypothetical case where ambient sound would be entirely removed from the floor. Consequently, the after-effect is stronger due to the presence of sound. Given that even a small increase in volatility strength in a given regime translates into a considerable increase in after-effect size^15^, the effect of sound may be detectable via comparing after-effect size with vs. without sound. While such a test cannot be implemented in the field for methodological and practical reasons, it can be implemented in the laboratory using controlled experimentation.

## Results

### Experimental design

To implement such a test, we built on the experimental design developed by Payzan-LeNestour, Balleine, Berrada, & Pearson^16^, which allows to measure the risk after-effect in a controlled laboratory task that mimics what professional traders experience in real-time on their terminal screens on a typical trading day. Task participants report their perceived risk/volatility of a medium-volatility financial asset following prolonged exposure to either a high-volatility asset (in half of the experimental trials) or a low-volatility asset (in the other half). The risk after-effect is quantified via the difference between the reported volatility of the medium-volatility stimulus post exposure to low volatility versus post exposure to high volatility.

We asked undergraduate students (mean age 21.6; 42% male) to perform sequentially two versions of the task in the laboratory, one with a sound element taken from a real-world trading floor, the other without, in a counterbalanced order [N = 47, within-subject design; a-priori power calculations conducted using G*power^17^ showed that this sample size gave us 90% power to detect a “medium” effect of sound (d = 0.5) using the test reported below to compare after-effect size with vs. without sound].

For each task version, in each experimental trial, the participants underwent a 50-s “adaptation period” that involved passively viewing a Brownian motion depicting trajectories of a financial asset displayed as a moving line-plot that was dynamically re-drawn on screen from right to left. A small dot signalled the start of the motion, which left a line as a visible trace of the dot’s vertical movement over time (see http://napubsound.weebly.com for demonstrations of the task). This stimulus, which we designed after consulting with finance professionals, closely mimics the kind of asset prices that real-world traders routinely track on their terminals.

There was a total of 20 experimental trials, for half of which the asset during the adaptation period had a high volatility (30%), whereas, for the other half, the asset had a low volatility (4%). Each adaptation period was immediately followed by a test phase in which we presented a medium volatility (10%) asset stimulus for 20 s. This timeline corresponds to a volatility regime and subsequent transition phase as depicted in Fig. 1: the adaptation period represents a volatility regime (i.e., an episode of prolonged exposure to extreme volatility); the test phase, the transition phase following this regime. Our parameter choice for volatility—30%, 4%, and 10% for high, low, and medium volatility—matches the levels observed in the field^15^. We systematically varied the mean level of the asset across all trials (for more details, see “Methods”).

**Figure 2:**
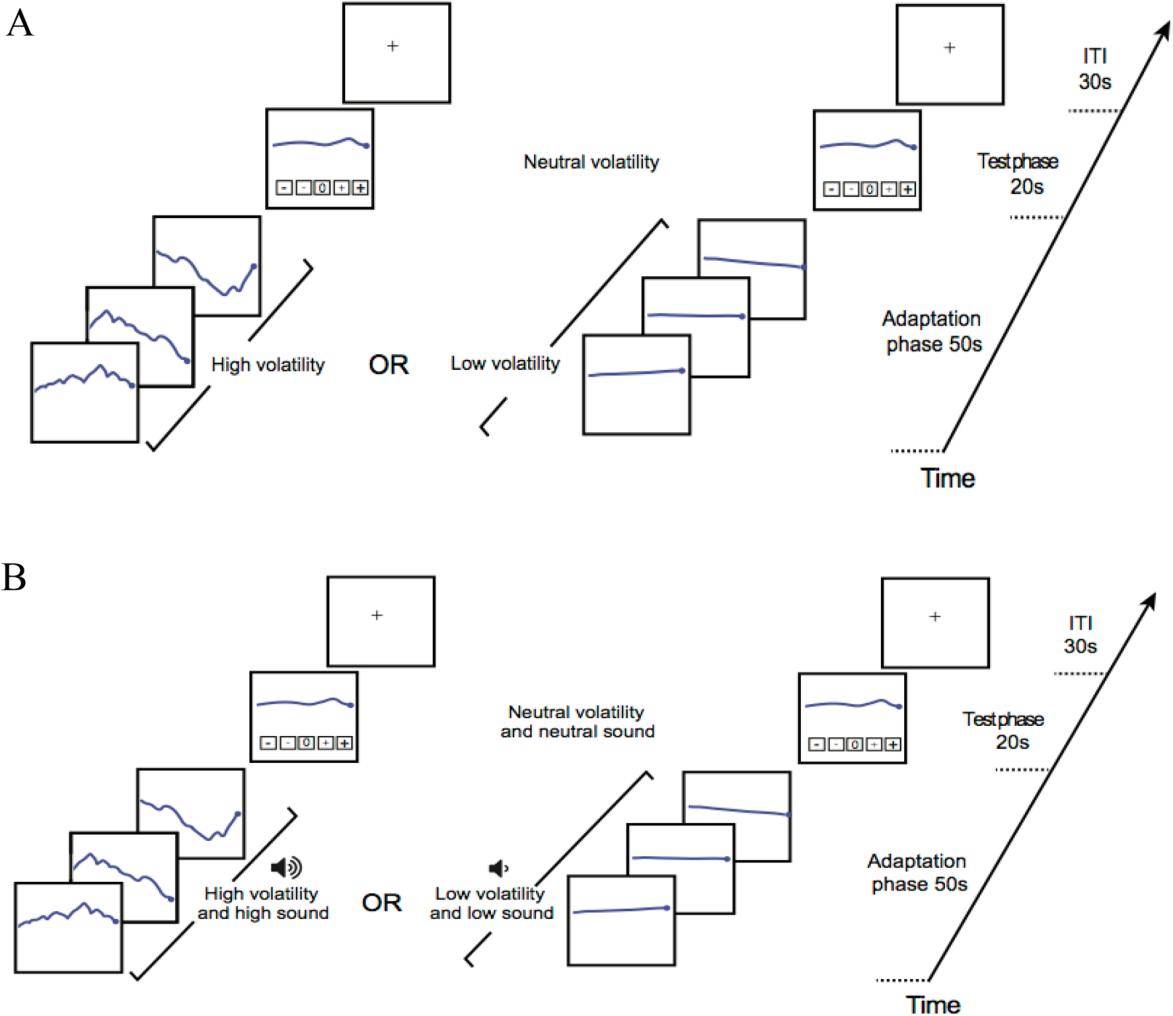
Timeline of an experimental trial of the task. (**A**) No sound treatment. Adaptation phase: Brownian motion depicting a financial asset with either high risk (30% volatility) or low risk (4% volatility) was displayed for 50 s. Test phase: medium (“neutral”) volatility was depicted by a 10% volatility Brownian motion displayed for 20 s, during which participants reported their perceived volatility on a 5-point scale using the mouse pointer. *ITI*: Inter Trial Interval. (**B**) Treatment with sound. That treatment was identical to the treatment without sound except that each volatility stimulus was paired with a congruent audio soundtrack recorded from a real-world financial environment.

In the task version with sound, we paired the viewing of each high-volatility stimulus with the presentation of a high-volume audio soundtrack recorded from a real-world turbulent financial environment. Likewise, we paired each low-volatility stimulus with a low-volume audio soundtrack recorded from a quiet real-world financial environment, and each medium-volatility stimulus was paired with a medium-volume audio soundtrack recorded from a neutral real-world financial environment (see Fig. 2 and “Methods”). The version without sound was devoid of any auditory stimulus and for each participant, the experimenter activated the noise canceling function of the headphones provided by the lab.

For each task version, in each trial, we asked the participants to rate the perceived volatility of the test stimulus on a 5-point scale using the mouse pointer (see Fig. 2 and http://napubsound.weebly.com). To avert decision biases potentially arising in rating paradigms, we used special task instructions whereby the participants could anchor their ratings to exemplar stimuli that we provided at the beginning of the task instructions in order to establish the meaning of the scale used in the experimental task. Participants saw 1-min videos of a very high/very low/medium volatility asset to explain what we meant by “very high volatility”/“very low volatility”/“medium or neutral volatility”.

We also provided all participants with high incentives to fully engage in the task. Specifically, we informed participants in the task instructions that the experimenters would monitor their focus and gaze on the computer monitor throughout the task using a webcam installed on top of the computer (visible to the subject). We further informed participants that they would receive a fixed payment of $50 only if they tracked all the stimuli displayed on screen (otherwise they would only receive a $5 show-up reward). During each session, we monitored participants’ behavior in a control room.

A justification for the two foregoing aspects of the design (pre-training task participants and providing them with significant monetary incentives) can be found in “Methods” and Payzan-LeNestour, Balleine, Berrada, & Pearson^16^. For the other aspects (in particular, why the current design gave us satisfactory power to test BRPH while seemingly simpler alternatives would not), see “Methods”. We also provide for replication purpose a video of the visual task instructions (as presented to the participants) and an audio file of the oral instructions that we recorded during one of the experimental sessions (available at http://napubsound.weebly.com).

## Results

**Figure 3:**
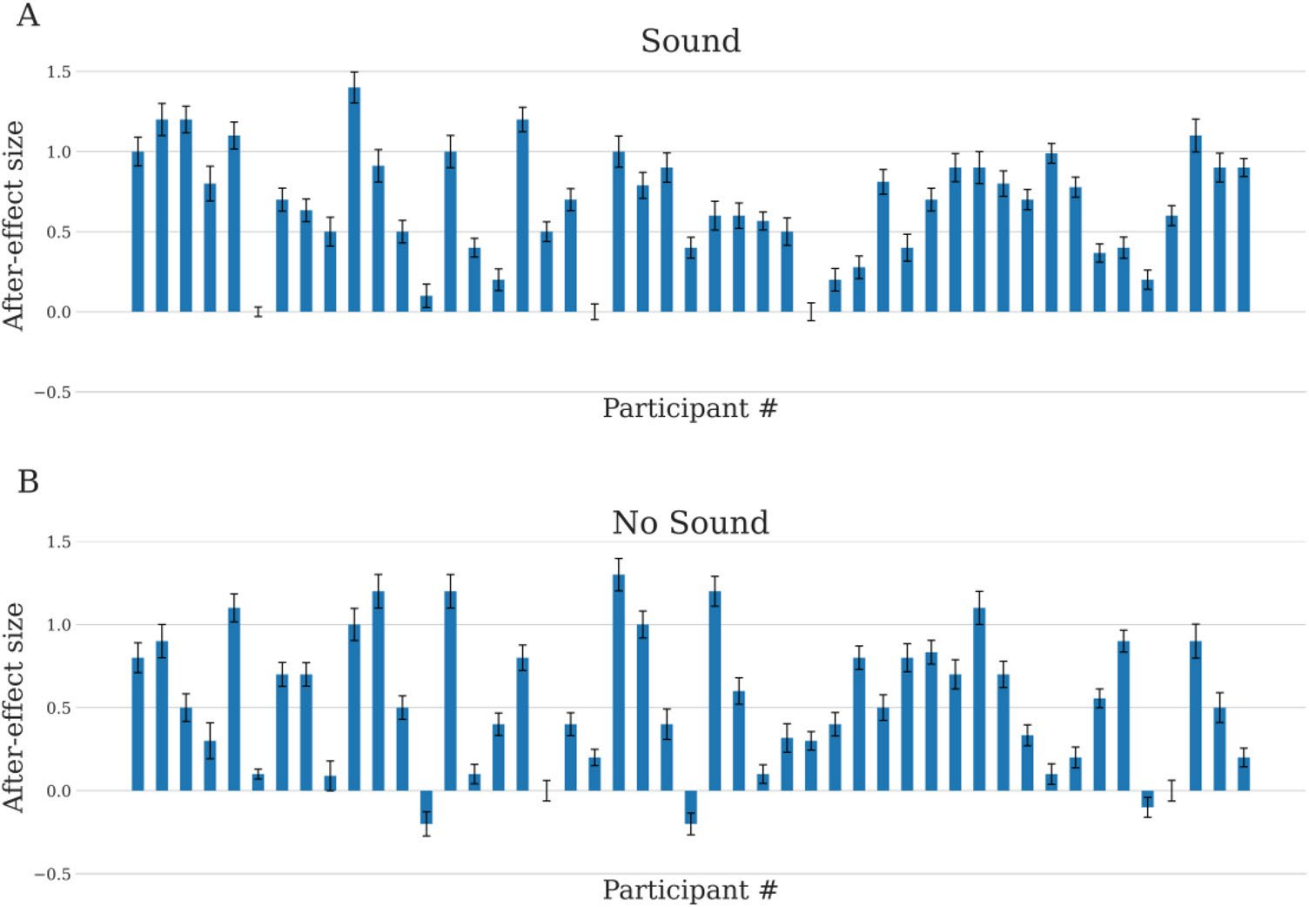
Risk after-effect for the experimental treatment without sound (**A**) and the treatment with sound (**B**). Each bar depicts the after-effect size for one of the participants (N = 47). Like in Payzan-LeNestour, Balleine, Berrada, & Pearson^16^, after-effect size was measured via the difference between the mean reported volatility post low (the reported volatility averaged across the trials in which the participant was exposed to low volatility in adaptation phase) and the mean reported volatility post high (the average reported volatility after exposure to high volatility). Error bars show standard error of the mean (sem).

We found a significant risk after-effect in the treatment without sound across task participants (Welch t-test: t = 4.40, P < 0.0001, one-sided; for more details, see “Methods”). The vast majority (94%) of participants featured the risk after-effect, i.e., they reported greater volatility after low-volatility exposure than after high-volatility exposure (Fig. 3A). In the treatment with sound, all participants displayed the risk after-effect (Fig. 3B). As predicted by BRPH, the after-effect in the treatment with sound was significantly greater than in the treatment without sound (paired t-test comparing after-effect size between the two treatments: t = 2.47, P = 0.018, two-sided; see Fig. 5A and Table 1 Column A).

**Table 1:** Comparison of after-effect size in the experimental treatment with sound (“sound”) vs. the treatment without sound (“no sound”), for each experiment.

	(A)	(B)	(C)
Mean after-effect size no sound	0.54 (0.40)	0.54 (0.41)	0.49 (0.37)
Mean after-effect size sound	0.67 (0.35)	0.72 (0.51)	0.60 (0.42)
Mean difference	0.13	0.18	0.11
95% CI	[0.03 0.24]	[0.06 0.30]	[− 0.02 0.23]
t-statistic	2.47	2.96	1.70
Cohen’s d	0.38	0.46	0.28
p-value	0.018	0.002	0.095
Observations	47	54	51

(A) First experiment. (B) Matched white noise experiment. (C) Crossover test. The table reports the magnitude of the after-effect averaged across all trials and participants (standard deviations in parenthesis). The mean difference (sound–no sound), as well as t-statistics, Cohen’s d and p-values of a 2-sided Welch t-test of the null hypothesis that the difference is null, are also reported.

### Additional analyses and experiments investigating underlying mechanisms

**Figure 4:**
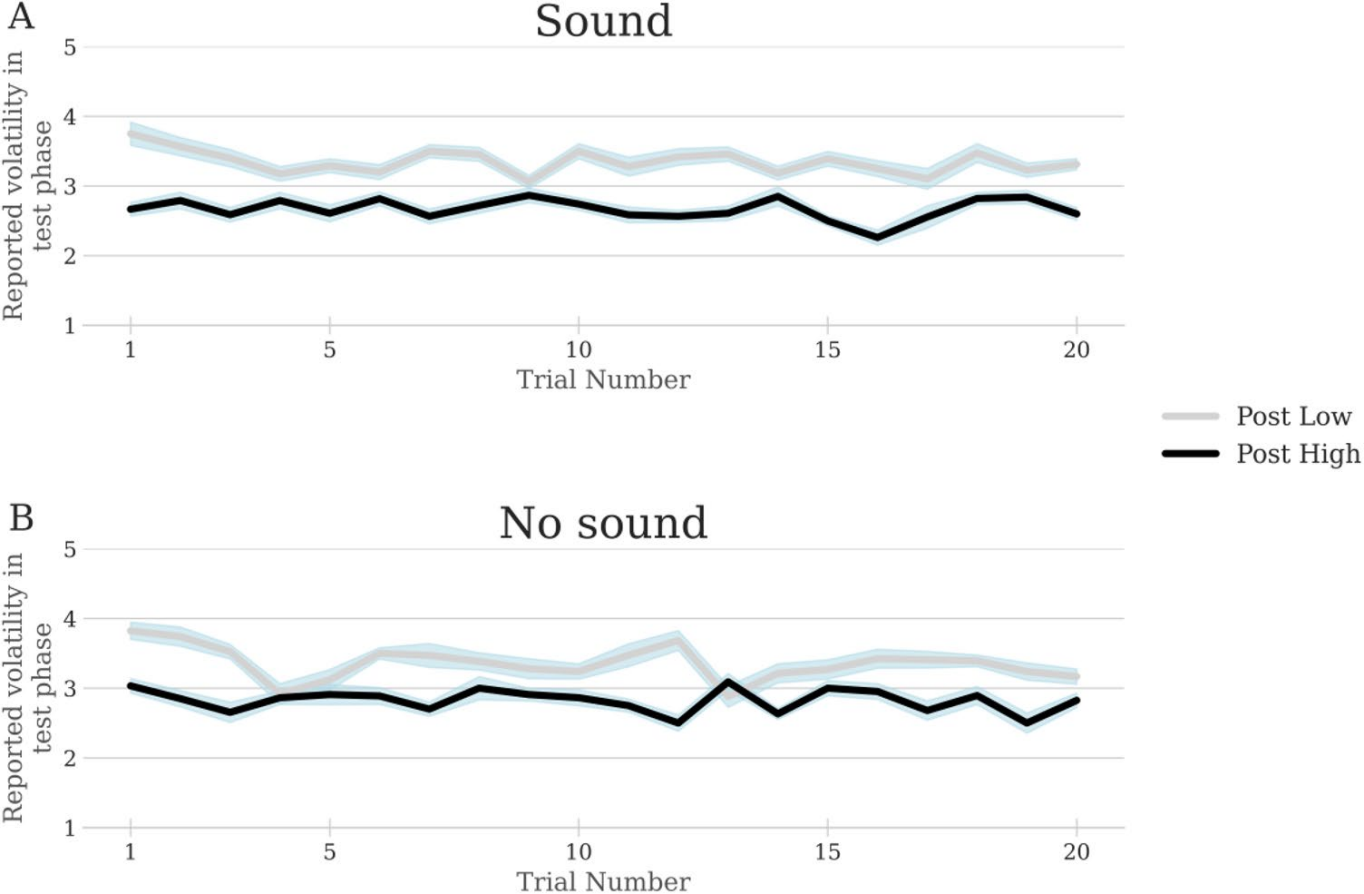
Time invariance of after-effect size in the treatment without sound (**A**) and the treatment with sound (**B**). The graph plots the average reported volatility on the scale (1: very low; 2: low; 3: medium; 4: high; 5: very high) for the medium-volatility test stimulus after exposure to high volatility (“post high”) vs. after exposure to low volatility (“post low”) at each trial. X-axis: trial number (1–28). Y-axis, “Post High”: Average reported volatility of the medium-volatility test stimulus across task participants who underwent high volatility in the adaptation phase of the corresponding trial; “Post Low”: Average reported volatility of the medium-volatility test stimulus across participants who underwent low volatility in the adaptation phase of the corresponding trial. For each trial, the difference between the post low and post high curves measures the size of the risk after-effect across participants. Shaded blue area shows standard error of the mean (sem).

We ran additional analyses and experiments to investigate the mechanisms underlying the effect of ambient sound on perceived volatility. First, we considered the possibility that participants learned to associate high (resp. low) auditory volume with high (resp. low) volatility throughout the task in the treatment with sound. To investigate the possibility that such learned association drove the effect of sound on volatility perception, we studied the temporal properties of the risk after-effect in the experiment. If the effect of sound on perceived volatility reflected the foregoing associative learning, the effect of sound should have been absent in the first trials of the task and would have emerged over time as the association between volatility intensity and sound intensity was acquired. Therefore, in the treatment with sound, the size of the risk after-effect should be significantly smaller in the first trials of the task (to reflect the absence of effect of sound) relative to the later trials (when the effect of sound is supposed to be present). Note that this reasoning holds only if after-effect size is time invariant in the treatment without sound, which turns out to be the case. There is no correlation between after-effect size and trial number in either treatment of the experiment (sound treatment: r(28) = − 0.0812; 95% CI = [− 0.4409 0.3010]; p = 0.6812; no sound treatment: r(28) = − 0.2266; 95% CI = [− 0.5529 0.1600]; p = 0.2463; see also Fig. 4). These findings are at odds with the associative-learning explanation for the effect of sound on volatility perception.

Next, we tested for the possibility that the influence of sound in the experiment was mediated by increased attention in the treatment with sound relative to the treatment without sound. The increased attention in the sound treatment could have been induced because the use of audio tracks from real-world financial environments made the experimental environment more “realistic” than in the treatment without sound. Making the experimental task more realistic might have helped participants remain more engaged and thus pay more attention throughout the task. As a result of the increased attention, the experimental data would be less noisy, i.e., statistical power would be greater, and the increased significance of the risk after-effect found in the treatment with sound could merely reflect this increased power.

To test for this possibility, we ran a follow-up “matched white noise” experiment which was identical to the first experiment, except that we altered the audio tracks used in the experimental treatment with sound [N = 54; power calculations conducted using G*power showed that this sample size gave us 95% power to detect a medium effect of sound using the foregoing two-sided paired t-test]. The audio tracks in the matched white noise experiment matched those used in the sound treatment of the first experiment for their main auditory properties, namely loudness and dynamic range, but the realistic component was removed by remixing their audio frequencies into pure white noise (all details for replication purposes can be found in “Methods”).

**Figure 5:**
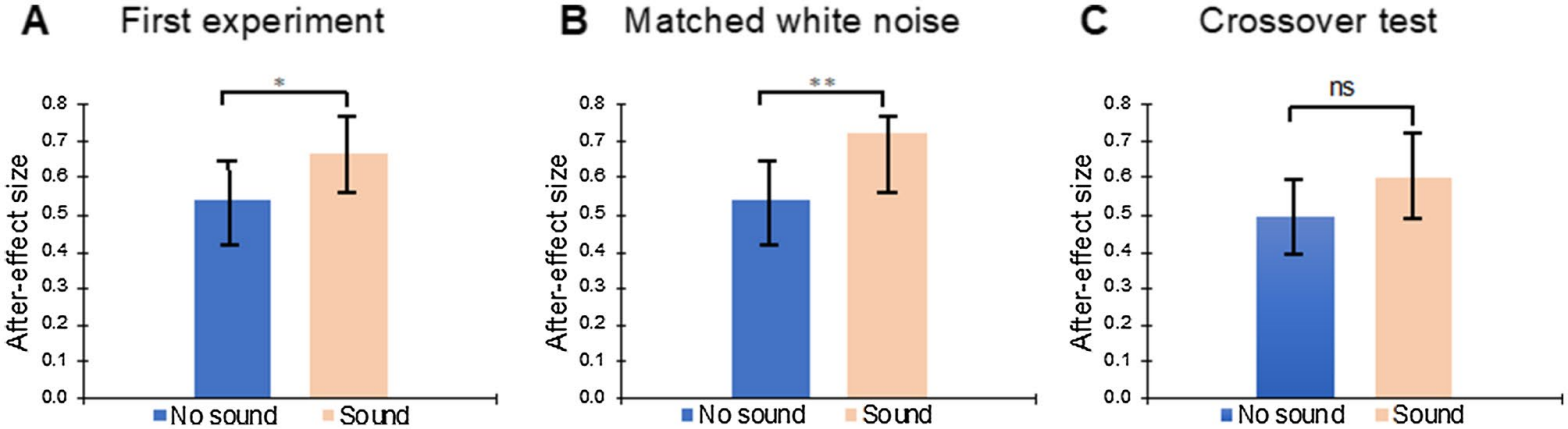
Difference in after-effect size between the treatment with sound (“sound”) and the treatment without sound (“no sound”), for each experiment. Y-axis: Mean after-effect size across participants (for the definition of after-effect size, see Fig. 3). *: difference is significant at p < 0.05; **: difference is significant at p < 0.01; *ns* difference is not significant at p < 0.05; see Table 1 for the statistics.

If it was the ‘real world’ nature of the audio tracks that enhanced the risk after-effect via increased attention, then the magnitude of the risk after-effect should not be increased in the treatment with sound compared to the treatment without sound in the matched white noise experiment. If anything, attention might be *decreased* in the treatment with sound inasmuch as white noise serves to distract attention^18^. However, if the effect of sound in the first experiment reflects the facilitatory and synesthetic congruency effects postulated by BRPH, it should still be present in the matched white noise experiment, where high (resp. low) volatility is systematically paired with high (resp. low) auditory volume, exactly like the first experiment. We found that the influence of sound on the risk after-effect was still present in the matched white noise experiment (Fig. 5B and Table 1, Column B), at odds with the increased attention explanation for the effect of sound in the experiment.

Taken together, therefore, the foregoing findings support BRPH. In the last stage of analysis, we aimed to investigate the relative importance of the arousal-related facilitation and synesthetic mechanisms postulated by BRPH. To examine whether the influence of sound on perceived volatility may work entirely through the former mechanism (facilitation), or whether it is more likely that both mechanisms combine, we took two steps. First, we studied participant response time. General facilitatory effects typically cause a *decrease* in response latencies^5,6^, whereas the synesthetic congruency effect is expected to cause an *increase*, as it requires auditory-visual integration, which is known to have a wide temporal window—it takes up to several hundred milliseconds to combine signals from different sensory systems into a single perceptual event^19^. We found that participant response time is higher in the treatment with sound (mean response time: 8.26 s) than in the treatment without sound (mean response time: 7.62 s). The difference in response time is significant according to a 2-tailed paired t test (t(100) = 3.23; p = 0.002; d = 0.25; for more details, see Sup Table 6 in “Methods”), which suggests that the effect of sound on perceived volatility goes beyond arousal-related facilitation.

Second, we ran a “crossover test” [N = 51, power calculations conducted using G*power showed that this sample size gave us 94% power to detect a medium effect of sound using the aforementioned two-sided paired t-test] in which we inverted the pairings of the auditory and visual stimuli used in the previous experiments in order to make the volume-volatility pairings incongruent. In the crossover test, high volatility was paired with *low* auditory volume, and low volatility was paired with *high* volume (for more details, see “Methods”). Apart from that feature, the experimental task used in the crossover test replicated the matched white noise experiment in all aspects.

We reasoned that if the sound effect observed in the first two experiments (i.e., after-effect size being larger in the treatment with sound than in the treatment without sound) was still significant in the crossover test, this would suggest that the foregoing synesthetic congruency effect is either absent or of second order relative to the facilitatory effect. For the congruency feature—pairing high (resp. low) volatility with high (resp. low) volume—is necessary to observe the synesthetic congruency effect, whereas it is irrelevant for arousal-related facilitation to occur.

The effect of sound seems to be reduced in the crossover test, in the sense that we were not able to reject the null hypothesis that after-effect size is the same in the sound vs. no sound treatments (Fig. 5C and Table 1, Column C). However, it is important to note that absence of evidence is not evidence of absence^20,21^, and we ran linear mixed regressions to further study the effect of sound in the crossover test and compare it to the effect in the previous experiments (see “Methods”). We found a significant effect of sound in the first experiment and the matched white noise experiment, whereas the effect is not significant in the crossover test; however, we could not conclude that it is significantly reduced in the crossover test.

Therefore, we view these findings as suggestive that an element of the influence of sound on volatility perception might have been lost when the visual and auditory signals were made incongruent, in line with the idea that the influence of sound on perceived volatility works through both general facilitation and synesthetic congruency effects combined, rather than general facilitation alone. But more work is needed to test this hypothesis (more in “Discussion”).

### Interviews with professional traders

To summarise, the experimental findings support the idea that by sharpening risk perception during volatility regimes, pairing the viewing of high (resp. low) volatility with high (resp. low) ambient sound worsens this same risk perception during the transition phase between two regimes. That is, ambient sound has an ambivalent effect on trader perception of risk, with both positive (within the regimes) and negative (during the transition between regimes) aspects.

In terms of “*behavioral interventions*”^22^, this ambivalence points to a trade-off where ambient sound is a key environmental variable to be fine-tuned to optimize trader perception. Whether sound is ‘friend’ or ‘foe’ for a given trader indeed depends on the trader’s trading style. For example, when focusing on momentum/market timing strategies, traders need to respond quickly to extreme market events, therefore they want their volatility perception to be optimized when the market moves into a new volatility regime; however, they do *not* need their perception to be optimized when the market moves into a transition phase. Hence overall it is optimal for traders to be exposed to ambient sound in this case. The opposite is true for contrarian/value strategies, which consist of trading against investor biases—investor sentiment and the like^23,24^. This kind of strategies requires quiet during trading to optimize the perception of volatility during the transition phase between regimes—which is when the opportunity set is greatest as one can potentially exploit the mispricing caused by the risk after-effect. In this case, being exposed to ambient sound implies a reduced capacity to take advantage of such mispricing.

To investigate if trading firms and their active traders are aware of the foregoing trade-off related to the ambivalence of ambient sound on trader decision making, we conducted interviews with professional traders. We asked them—without mentioning the topic of our study, to avoid biasing their answers—why traders sit so close to one another although space is not limited (so they could choose to be spread out). Their replies included: *“We like the traders to feel the buzz, to hear the excitement when opportunities are present*.” [interview of the head of trading at a market making firm based in Sydney]; “*The buzz does help keep you engaged, so hearing others is helpful on the margin.”* [interview of a proprietary trader based in New York]*; “He* [referring to a star trader who had chosen to leave the firm to trade by himself and came back after 6 months of flat performance] *told us that he missed the energy and sound of being on the floor*.” [interview of the CIO of a large proprietary trading firm based in New York]; “*On a trade floor, you live in a carefully calibrated sound field. Overall, there was a buzz from each area on the floor. If you heard some noise from FX, you quickly checked that sector*.” [interview of a former trader in a large Swiss bank]; “*There are lots of times when you can kind of feel the buzz on market moves […] Most of the time floor is slow so you definitely focus more when you feel it hopping*.” [interview of a floor broker in New York].

These interviews data suggest that professional traders mostly intuit the positive aspects of the influence of sound on risk perception, while missing the negative aspects. This leads them to unconditionally ‘seek the buzz’ and may help explain why they choose to sit close to one another (although they cannot remove their visual focus from the multiple screens they concentrate on and hence rarely talk to each other in practice); and also why the modern day-trading floor layout and design in proprietary trading firms and banks resemble the trading floors of the 1980s and earlier, with a cluster of traders seating together on an open floor plan (which seems anachronic viewed from the general movement towards entirely suppressing trading floors from exchanges).

## Discussion

Taken together, the current findings suggest that ambient sound can influence the visual perception of risk in professional traders due to both general facilitation—consistent with prior evidence for the involvement of the locus ceruleus-norepinephrine system in uncertainty appraisal^25^, and possibly a general association between auditory volume and visual instability. The latter might have an evolutionary origin if using both visual and auditory cues to establish the relative predictability of events provides an advantage over using either modality alone^26–28^.

As such, the present study adds to a rich body of work showing cross-modal links between audition and vision in visual tasks^1,3,19,29^. Our findings likewise argue against traditional modality-specific accounts of separate channels for auditory and visual information processing^30^ and support the claim that different sensory modalities influence one another in decision making.

Our findings also echo recent applied research that has suggested exploiting pre-existing (hardwired) mental associations between a given auditory stimulus and a corresponding visual event when designing auditory warnings for use in the cockpit to inform aircrew about dangerous conditions, such as low altitude or the presence of a military threat^31^. For example, it has been proposed that displaying the cry of a bird of prey to signal the presence of an enemy jet fighter may work because of a hardwired association between the two stimuli (both fly and have attacking features, so the cry stimulus represents the visual event of the enemy jet fighter). One distinctive aspect of the association between ambient sound with visual volatility proposed in this study is that it is not referential—what matters is the level of volatility and loudness per se irrespective of stimulus content. This is reminiscent of the early ergonomic alarm design literature which focused on the alerting capacity of sounds rather than their informational nature^6^ and contrasts with recent work primarily focused on the type of ambient sounds^32,33^.

Our study leaves us with many avenues for future research. One important question concerns interindividual differences in the effect of sound on volatility perception. It is clear that the effect of sound documented here does not present itself consistently across participants (see supplementary information). We speculate that interindividual differences in factors influencing attentional focus and emotional arousal may explain why some individuals are susceptible to the effect while others are not. It could also be that the effect is of a larger magnitude in populations of older adults, as prior studies suggest^34,35^.

More work is also needed to understand how finely people can discriminate between specific acoustic features of their environment to assess risk. In this study, we focused solely on the decibel level of a given context. But in light of the degree of complexity and sophistication of the auditory systems used by other species to signal and detect risk^36,37^, one may expect humans to use other acoustic features to assess the riskiness of their environment. Besides decibel levels, may they use features such as high-energy peaks above given decibel thresholds and spanning wide bandwidth, as seems to be the case in birds^38^?

Future research may also assess the relative contribution of the foregoing facilitatory and synesthetic effects to the influence of sound on risk perception, and to examine other—not mutually exclusive—possible mechanisms. Process data (e.g., eye-tracking, neural measurement) could be used to that purpose, following the lead of the latest neuroscientific work in multisensory perception^39^.

No matter what the underlying mechanisms turn out to be, the influence of sound on risk perception evidenced in this study has high ecological relevance for the financial industry. Prior finance research showed that the magnitude of ambient sound has unique informational content for trader decision-making^40^. The present research suggests that the role of sound in trader decision-making goes beyond information exchange and further consists of helping professional traders “feel risk”^12^ during extreme volatility episodes, with ambivalent effects on their overall perception of risk. Optimizing trader risk perception is important both from individual and market-wide perspectives because, despite the increasing automation in the mechanical aspects of trading in many modern financial markets, human traders continue to perform tasks that involve judgement, such as key trading decisions, forecasting future prices, etc. Such judgment tasks, which are ultimately what determine price levels, hinge on the accuracy of traders’ risk perception.

That said, it is worth emphasising certain limitations of our study, related to ecological validity. First, sound-induced facilitatory effects of the kind evidenced in this study may be stronger in the field than in the current experimental paradigm. In the field, multitasking typically prevails, implying a decreased attentional focus to any specific task, and hence an increased effect of attentional facilitation^35^. In contrast, our task participants were highly focused on the risk judgment task at hand by design, mechanically implying a reduced importance of facilitatory effects. Second, it could be that the magnitude of the effect of sound differs if the sound emanates from the environment, rather than being presented over headphones as was the case in our experimental paradigm. Evidence indeed suggests that on occasion, people respond differently as a function of whether the auditory stimuli are presented over headphones versus from external loudspeakers^41^, consistent with the idea that auditory stimuli presented close to the head (i.e. in peripersonal space) are more arousing than stimuli presented further from the head^35^.

Perhaps the main merit of the current study, from an applied perspective, is to bring to the attention of professional traders the possibility that ambient sound influences their volatility perception in an ambivalent manner. Our interview data indeed suggest that professional traders are only aware of the positive influence of ambient sound on their decision-making. In the same way that prior reports of stock market “anomalies” helped traders modify their decision-making^42^, could it be that dissemination of the current line of research will put professional traders “on their guard” for possible biases in their perception of volatility? Some have indeed pointed out that while perceptual biases of this kind are impossible to intuit, once people are made aware of their possibility, they can consciously correct their decision-making^43^.

The idea that professional traders face a trade-off—between improving risk perception during volatility regimes versus during the transition phases between regimes—echoes recent applied research into the optimal use of auditory warnings in complex environments such as extensive care units and military cockpits^31,44,45^. For example, Meredith and Edworthy^45^ stressed that users of auditory warnings on medical equipment face a similar trade-off, in that case between avoiding information overload and distraction (if using too many warnings) versus avoiding underutilizing important information sources (if using too little). It was further stressed that the solution to the trade-off is context-dependent (it depends on the decision maker’s state of vigilance and stress among other factors), as in the trading case (the solution to the trade-off depends on the trader’s current trading strategy, as described above). Whether this analogy between the medical and trading decision-making domains may extend to other work settings is an open question for future research.

Finally, we highlight an important avenue for future research on the impact of ambient sound on risk taking, to complement the current line of work. The latter focuses on the channel of risk *perception*. The risk *preferences* channel is equally important: recent work points to the possibility that ambient noise increases risk appetite for a given level of perceived risk^46^. The risk perception vs. risk preferences channels should be disentangled and studied separately, in the vein of the latest research in social science^47,48^.

## Methods

### Participants

Participants (N = 47 in the first experiment; N = 54 in the follow-up “matched white noise” experiment; N = 51 in the final experiment) were undergraduate students from *The University of New South Wales* (UNSW) recruited through Orsee (mean age 21.6, median 20; 42% male). All experiments were performed in accordance with relevant guidelines and regulations and approved by *UNSW Ethics Panel*. Informed consent was obtained from all participants.

### Apparatus

All experiments were performed in a darkened room on computer monitors with a resolution of 1920 × 1080 pixels, with a frame rate of 60 Hz. The experimental programs are web applications custom coded in PHP.

Each participant performed a total of 28 trials, 20 experimental, 3 diversion and 5 control trials, all randomly interleaved. The asset stimulus was a Brownian process which mean value was varied across trials; the mean values that we used are 0, ± 0.1, ± 0.2, ± 0.3, ± 0.4 (in randomized order). In the experimental trials, volatility was set as follows: for each mean value, there was one “high-volatility trial” featuring a 30% volatility asset in the first phase of the trial followed by medium (10%) volatility in the test stimulus, and one “low-volatility trial” featuring a 4% volatility asset in the first phase of the trial followed by medium (10%) volatility in the test stimulus. The diversion trials consisted of medium volatility followed by either high or low volatility in the test stimulus, and the control trials consisted of a medium-medium transition.

In the sound treatment of the first experiment, each asset stimulus on the screen was paired with an audio soundtrack recorded from either a trading pit, a stock exchange trading floor, or an office space, broadcasted over TDK NC-200 headphones (we disabled the noise cancelling feature). Visual and auditory stimuli were matched as follows. For each category of auditory stimulus (high/low/medium), we created a pool with 20 recordings extracted from one of three different financial environments (trading pit, stock exchange, office space). Each high (resp. low) volume recording was extracted from a turbulent (resp. quiet) financial environment; each medium recording was extracted from a neutral financial environment. We adjusted the volume to ensure that all high-volume soundtracks were between 90 and 100 dB, low volume soundtracks were between 20 and 30 dB, and medium volume between 60 and 70 dB. Our volume-volatility pairings were congruent, in the sense that for each trial one of the recorded sound tracks was randomly selected from the relevant pool: each high-volatility stimulus was paired with one of the high-volume recordings, each low-volatility stimulus was paired with one of the low-volume recordings, and each medium-volatility stimulus was paired with one of the medium-volume recordings.

The audio tracks used in sound treatment of the matched white noise experiment were generated by shuffling the audio tracks used in the sound treatment of the first experiment. Each sample in the shuffled waveform was a random sample from a window ± 50 ms around that time point in the unshuffled waveform. This allowed us to preserve the waveform statistics of the original audio tracks while removing any meaningful content (e.g., human voices, typing sounds etc.) from them. So by design, the sound treatment of the matched white noise experiment used the exact same pairing of visual and auditory stimuli as the one used in the first experiment; the only difference between the two experiments is that any meaningful content from the original audio tracks was removed so that the audio tracks of the follow-up experiment are pure white noise.

In the final experiment (“crossover test”), the pairing of visual and auditory stimuli was inverted compared to that in the previous experiments in order to make the volume-volatility pairings incongruent: each high-volatility stimulus was paired with one of the low-volume recordings, each low-volatility stimulus was paired with one of the high-volume recordings (and each medium-volatility stimulus was paired with one of the medium-volume recordings, like before).

For 24 of the participants in the first experiment, the experimenters mistakenly set the high volatility parameter to only 25% (it was supposed to be 30% as for all the other participants in all three experiments, as indicated above). This mistake likely explains the smaller effect of sound in the first experiment relative to that in the matched white noise experiment (see Table 1 of the paper). It precludes comparing the magnitude of the effect of sound between the first and matched white noise experiments. However, such a comparison is *not* part of testing the increased attention hypothesis reported in the main text. The increased attention hypothesis indeed predicts that the effect of sound should be absent or negative in the matched white noise experiment. Therefore, our finding of a significantly positive effect of sound in the matched white noise experiment (as documented in the main text) is sufficient to falsify that hypothesis.

### Procedure

The reader will find on the website http://napubsound.weebly.com/ the following material which we believe is important for replication purposes:

- The online task instructions as the task participants saw them, for each experiment;
- An audio file of the oral instructions recorded during one of the experimental sessions; these oral instructions were provided at the end of the instruction phase of each experimental session, just before the task participants performed the task;
- Demos of the task used in each experimental treatment of each experiment.

In the recruitment email used for each experiment, potential participants were told they would participate in two experimental sessions conducted one week apart. Registration had to be completed for both experimental sessions. Half of the participants were exposed to the experimental treatment without sound first and to the treatment with sound second; the other half were exposed to these treatments in the reversed order.

Upon arrival at the lab, the participants were asked to read the online task instructions and were encouraged to ask any clarifying questions to the experimenter. To avert well-known decision biases related to the use of rating scales (e.g., subjects using the stimulus seen in the adaptation phase as a reference point to rate the test stimulus), the task instructions began by pinning down the meaning of the volatility scale used in the task via exemplar stimuli that defined the two extreme points on the scale (1: very low; 5: very high) and the middle point (3: medium). Specifically, we showed a 1-min video of a very high / very low / medium volatility asset to explain what we meant by “very high volatility” / “very low volatility” / “medium or neutral volatility.”

We provided participants with high incentives to fully engage in the task. Specifically, participants were informed in the task instructions that they would receive a fixed payment of $50 only if they tracked all the stimuli displayed on screen (and also kept the headphones on in the experimental treatment with sound), otherwise they would only receive a $5 show-up fee. The subjects received a reminder of this condition immediately prior to each experimental session. During each session author EPLN and 1 assistant monitored each subject's behavior in a control room.

Just before performing the task, participants were re-explained the distinctive nature of the task; the need to balance high pace of reply while never replying randomly was particularly emphasized by the experimenter (participants were warned that after a few missed trials the task would stop automatically). Participants were also reminded that their behavior would be recorded by a webcam (installed on top of computer; visible to the subject) throughout the task and that they had to keep their focus and gaze on the monitor during the entire experiment in order to get the $50 payment. Each experimental session took approximately 90 min overall (the task itself took 45 mins).

All the experiments documented in this study were performed in accordance with relevant guidelines and regulations approved by the ethics panel at the University of New South Wales and written consent was obtained from all participants. The experimental data are available at https://osf.io/vg4zk/. Demos of the main experimental task, a video of the visual task instructions (as presented to the participants) and an audio file of the oral instructions that we recorded during one of the experimental sessions, are available at http://napubsound.weebly.com.

### Rationale for the current design

The first strategy that comes to mind to test the *Bimodality of Risk Perception Hypothesis* (BRPH), namely comparing judgments of extreme volatility with vs. without sound, would be problematic for applied purposes as it would give an incomplete view of the effect of sound on trader risk perception (it would be silent on the effect of sound on the perception of medium volatility during the transition phases—as described in the main text). Besides, it cannot work in the current setting owing to ceilling and floor effects (i.e., very high volatility stimulus rated ‘5’ and very low volatility rated ‘1’ both in the presence and absence of sound, even if actual perception does differ across the two treatments). The difference is not measurable because the 5-point scale used in the current paradigm is too coarse. Using a finer scale (e.g., 10-point scale) is not a solution as participant ratings are then too noisy, thereby requiring an unachievably large sample size to get a chance to detect the effect. (Increasing sample size beyond a few hundreds of participants is unachievable given budgetary constraints and the high cost of the experiment per participant ($100, which is required to test BRPH for the reason explained in the next paragraph). Comparing after-effect size with and without sound has more power for the reason stressed in the main text (it leverages the fact that after-effect size increases non-linearly with extreme volatility strength).

It is similar power considerations that led us to use the foregoing incentive device to ensure that participants were highly incentivized to remain fully engaged throughout the task. We reasoned that, given the repetitive nature of the task, incentivizing participants the way we did was essential to ensure high-quality in participants’ risk perception reports.

Our motivation for training participants in the task instructions by showing them exemplar stimuli for each volatility category (high/medium/low) was to avert decision biases that commonly arise in rating paradigms. Specifically, we wanted to ensure that participants’ rating of the test stimulus in a given trial would *not* use the stimulus seen in the exposure phase of the trial as an anchor or ‘reference point’. Rather, if our strategy worked, the examples seen during the task instructions would be used as such reference points. To verify that this strategy worked, we checked that in the diversion trials, the very high volatility and very low volatility test stimuli were rated 5 and 1 respectively, barring a few exceptions (see “Exclusion Criterion” next). Note that other decision biases such as assimilation/priming bias participant judgment in the *opposite* direction to the risk after-effect. [Assimilation/priming indeed leads participants to judge the medium-volatility test stimulus as more volatile after a volatile stimulus and flatter after a flat stimulus.] Therefore they’re not a confounding factor for our measurement of the risk after-effect (see Ref.^16^ for more details).

### Exclusion criterion

In the first experiment, three participants failed to comply with the instruction rules (they almost fell asleep during the session). Their data were therefore discarded (and they only received the $5 show-up fee). We also discarded the data from one subject who did not respond appropriately on the diversion trials (test stimulus—which by design featured extreme volatility, as explained above— was rated as medium). In the matched white noise experiment, we had to discard the data from two participants who likewise failed to comply with the rules. In the crossover test, we discarded the data from one participant who provided absurd replies to all the diversion trials (test stimulus was rated as medium). We also had to discard the data from a couple of participants who did not show up at the second experimental session (first experiment: 2 participants; crossover test: 1 participant).

### Statistics

#### Measure of the risk after-effect

For each participant, the risk after-effect was quantified via the difference between the mean reported volatility “post low” (the reported volatility averaged across the trials in which the subject was exposed to low volatility in the adaptation phase) and the mean reported volatility “post high” (the average reported volatility after exposure to high volatility). To assess the significance of the after-effect across participants in each of the experimental treatments, we ran one-sided Welch t tests (null hypothesis: subjects’ perceived volatility post low was on average lower or equal to its level post high). Sample size equals the number of subjects. The main statistics of interest are reported in Sup Tables 1–3.

#### Measure of the sound effect

To test the conjecture that the risk after-effect is amplified by sound, we ran a 2-sided paired t-test based on the individual differences in after-effect size in the sound vs. without sound experimental treatments (null hypothesis: the magnitude of the after-effect is the same in both treatments). The null hypothesis is rejected at the 5% threshold in the first experiment and at the 1% threshold in the matched white noise experiment. It cannot be rejected in the final experiment (“crossover test”). See Table 1 in the main text.

We also ran linear mixed effects regression models with trial-wise after-effect (report post-low—mean report post-high) as the outcome. (The main conclusions were unchanged when (mean report post-low—report post-high) was used instead as the outcome.) The fixed effects were a dummy variable for condition (sound vs. no sound), Box-Cox transformed response time, age, gender, and by-participant random intercepts nested in condition. We found a significant effect of sound in the first experiment and the matched white noise experiment but not in the crossover test (Sup Table 4, regressions (1–3)). To test whether the effect of sound is significantly reduced in the crossover test, we set up a linear mixed model with a variable comparing the first experiment and the matched white noise experiment pooled together to the crossover test (Sup table 4, regression (4)). Our main variable of interest was the interaction between that variable and the dummy variable for condition (sound vs no sound). Finding a significantly negative coefficient for that interaction would be evidence that the effect of sound was significantly reduced in the crossover test. We found a negative coefficient, though not significant, possibly due to a lack of power—we only had 70.7% power to detect a medium effect size and 16.1% to detect a small effect. Power was assessed using the simglm package in R by simulating 1000 datasets using model weights from the regressions as effect sizes with a specified a priori effect size (small/medium) for the interaction. We checked if the interaction was significant for each simulated dataset, and measured power as the proportion of significant outcomes across the 1,000 datasets. Note that the main conclusions were unchanged but power was even lower in a specification of our linear mixed model in which the dependent variable was the trial-wise metric (report—mean report in the control trials) and the fixed effects included a dummy variable for Trial type (high volatility vs low volatility), the interaction Trial type × Condition, and the triple interaction Trial type × Condition × Experiment (our main variable of interest, with which to assess whether the effect of sound is significantly decreased in the crossover test).

#### Response time and risk after-effect

To study potential relation between size of the risk after-effect and response time in the experiment, we ran the following regression for each experimental treatment (sound and without-sound):

1 $${AAE}_{i}=\alpha +\beta { \times ART}_{i}+{\varepsilon }_{i}$$

where the variables are: ${AAE}_{i}$ = after-effect size (as defined in the main text: gap between average reported volatility post low and average reported volatility post high) for subject *i, *${ART}_{i}$ = average response time for subject *i* defined as follows:

$${ART}_{i}=\frac{{ARTL}_{i}+{ARTH}_{i}}{2},$$

where, ${ARTL}_{i}$ = average response time after exposure to low volatility for subject *i*; ${ARTH}_{i}$= average response time after exposure to high volatility for subject *i*.

Sup Table 5 reports the results for the first experiment (the results are qualitatively the same for the other experiments; not shown for space reasons but available on request). The $\beta$ coefficient is not significant, which suggests that there is no relation between after-effect size and response time across participants (replicating the findings^16^).

## Supplementary Information

Supplementary Information
