# Supplementary material for: Impact of ambient sound on risk perception in humans: neuroeconomic investigations
Source: Sci Rep. 2021 Mar 8;11:5392. doi: 10.1038/s41598-021-84359-7 (PMC7940636; doi:10.1038/s41598-021-84359-7)

Electronic Supplementary File for: "Impact of ambient sound on risk perception in humans: neuroeconomic investigations"

Elise Payzan-LeNestour[1]*, Lionnel Pradier[1], James Doran[1], Gideon Nave[2], & Bernard Balleine[3]

1. University of New South Wales Business School
2. The Wharton School of the University of Pennsylvania
3. University of New South Wales, School of Psychology

* Correspondence to: elise@unsw.edu.au; ORCID: https://orcid.org/0000-0001-8658-3208

Sup Table 1. Reported volatility for the medium-volatility test stimulus in the experimental treatment without sound ("no sound") and the treatment with sound ("sound") in the first experiment. Mean reported volatility in the test phase after exposure to low volatility ("post low") and after exposure to high volatility ("post high") in the two experimental treatments (standard deviations in parenthesis). For each treatment the mean difference (post low - post high), as well as t-statistics and p-values of a Welch t-test of the null hypothesis that the difference is null, are also reported.

	No sound	Sound
Mean post low	3.38	3.37
	(0.63)	(0.50)
Mean post high	2.84	2.70
	(0.55)	(0.46)
Mean difference	0.54	0.67
t-statistic	9.11	13.17
p-value	0.000	0.000
P5	0.42	0.57
P95	0.65	0.77
Observations	47	47

Sup Table 2. Reported volatility for the medium-volatility test stimulus in the experimental treatment without sound ("no sound") and the treatment with sound ("sound") in the matched white noise experiment. See Sup Table 1 for legend.

	No sound	Sound
Mean post low	3.18	3.29
	(0.57)	(0.60)
Mean post high	2.64	2.57
	(0.52)	(0.57)
Mean difference	0.54	0.72
t-statistic	9.64	10.36
p-value	0.000	0.000
P5	0.43	0.59
P95	0.65	0.86
Observations	54	54

Sup Table 3. Reported volatility for the medium-volatility test stimulus in the experimental treatment without sound ("no sound") and the experimental treatment with sound ("sound") in the final experiment ("crossover test"). See Sup Table 1 for legend.

	No sound	Sound
Mean post low	3.18 (0.54)	3.23 (0.55)
Mean post high	2.69 (0.56)	2.63 (0.46)
Mean difference	0.49	0.60
t-statistic	9.56	10.29
p-value	0.000	0.000
P5	0.39	0.49
P95	0.60	0.72
Observations	51	51

Sup Table 4. Regression outputs for the linear mixed model estimating after-effect (post-low – mean post-high). “Condition (sound)”: dummy to compare the no sound treatment (reference) vs. the sound treatment. “Experiment (crossover)”: dummy to compare the first experiment and the matched white noise experiment (reference group) vs. the crossover test. All continuous variables (after-effect, RT, age) were z-scored. Response time was Box-Cox transformed. (1) First experiment (2) White noise experiment (3) Crossover test. (4) Interaction model (includes the data from all three experiments). ***, **, and * denote coefficients significant at $p<0.001$, $p<0.01$ and $p<0.05$ respectively.

	(1)	(2)	(3)	(4)
(Intercept)	.069 (.048)	.029 (.039)	.001 (.040)	.034 (.197)
Condition (sound)	.089 (.041) *	.112 (.033) **	.033 (.039)	.099 (.026) ***
Response Time	-.057 (.022) **	-.067 (.019) ***	-.038 (.018)	-.054 (.011) ***
Age	.014 (.031)	.001 (.027)	.016 (.022)	.013 (.015)
Gender (male)	.012 (.062)	.014 (.056)	.086 (.046)	.051 (.030)
Experiment (crossover)	-	-	-	-.020 (.041)
Condition x Experiment	-	-	-	-.060 (.046)
R^2	8.5%	7.6%	5.4%	7.3%
Observations	1860	2136	2014	6010

Sup Table 5. Regressions testing for a potential relationship between after-effect size and reaction time in the first experiment. Reaction time was Box-Cox transformed ($\lambda = 0.18$) to reduce skew. Coefficient estimates from Regression (1) in Methods for the experimental treatment without sound (left column) and the treatment with sound (right column) in the first experiment. T-statistics (in parenthesis) were calculated with heteroskedasticity robust standard deviations. ***, **, and * denote coefficients significant at $p<0.01$, $P<0.05$ and $p<0.10$ respectively.

	No sound	Sound
β	0.131	-0.019
	(0.863)	(-0.141)
α	0.210	0.713**
	(0.548)	(2.136)
R^2	1.63%	0.04%
Observations	47	47

Sup Table 6. Comparison of response time in the experimental treatment with sound ("sound") vs. the treatment without sound ("no sound"), for the first and matched white noise experiments. The table reports descriptive statistics for response time (Box-Cox transformed), averaged across all experimental trials and participants. The mean difference (sound – no sound), as well as t-statistics, Cohen's d and p-values of a 2-sided paired t-test of the null hypothesis that the difference is null, are also reported.

	**Sound**	**No sound**
**Mean**	2.48 (0.46)	2.37 (0.45)
**SD**	2.52	2.42
**Median**	1.52	1.34
**Min**	3.67	3.39
**Max**	0.06	-0.20
**Skew**		
**Mean difference**	0.12	
**95% CI**	[0.04 0.19]	
**t-statistic**	3.23	
**Cohen's d**	0.25	
**p-value**	0.002	
**Observations**	101	

Sup Fig 1. Points show the difference in after-effect (sound – no sound) for participants in each experiment. Error bars show standard error of the mean (sem). (A) First experiment. (B) Matched white noise experiment. (C) Crossover test.

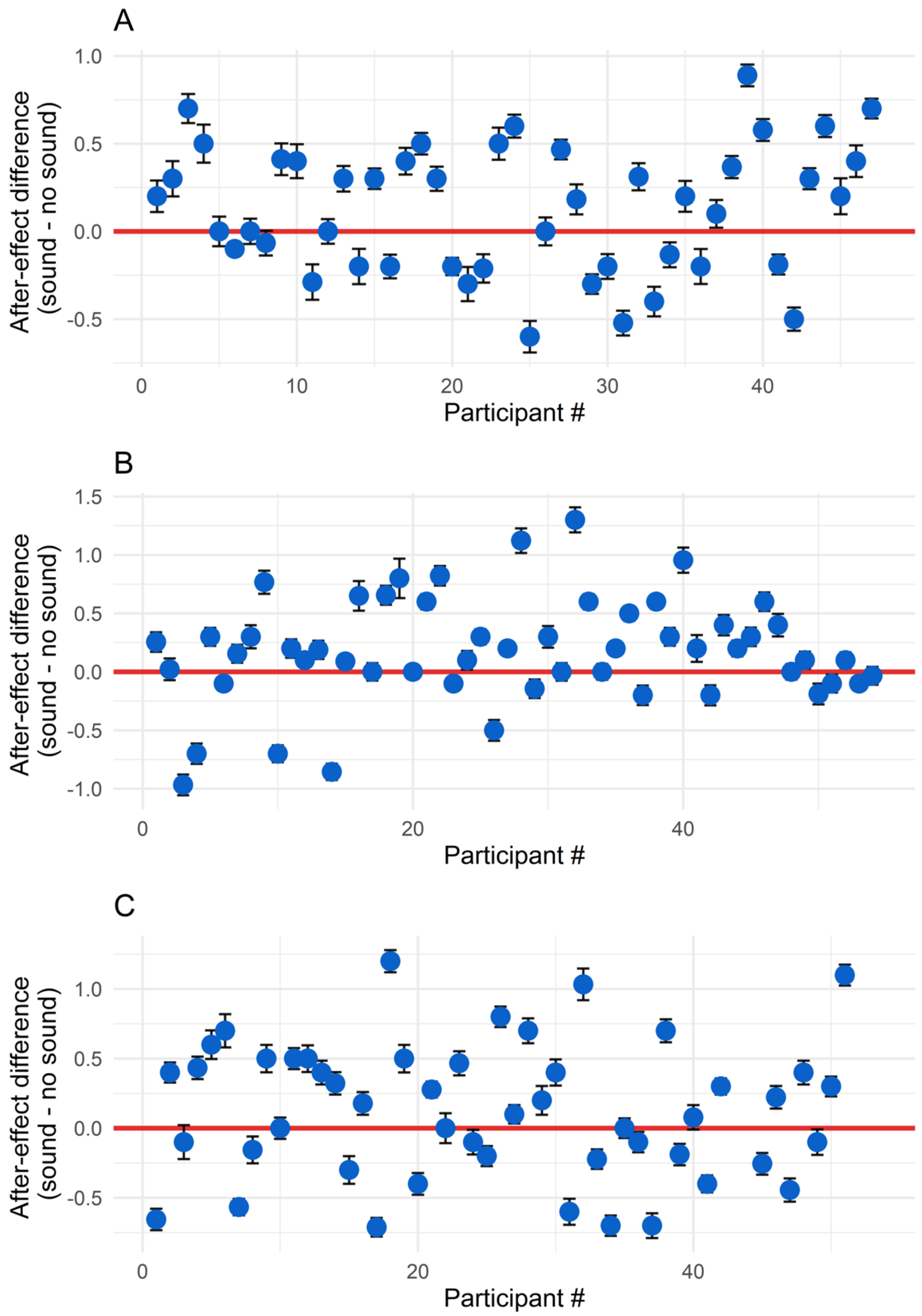

Supplement: Supplementary file 1 — Supplementary Information [file 41598_2021_84359_MOESM1_ESM.pdf]
